# Gene variation and population structure of *Pampus chinensis* in the China coast revealed by mitochondrial control region sequences

**DOI:** 10.1080/23802359.2021.1878963

**Published:** 2021-07-06

**Authors:** Peng Sun, Jiayue Yu, Baojun Tang, Zhidong Liu

**Affiliations:** a Key Laboratory of East China Sea Fishery resource Exploitation, Ministry of Agriculture, East China Sea Fisheries Research Institute, Chinese Academy of Fishery Sciences, Shanghai, China; b College of Fisheries and Life Sciences, Shanghai Ocean University, Shanghai, China

**Keywords:** Control region, genetic structure, mitochondrial, fishery management

## Abstract

*Pampus chinensis* is a commercially important fishery species in the Indo-West Pacific region. In the present study, the genetic variation of *P. chinensis* among 10 sampling localities along the China coast and one from the Indonesia region was evaluated using mitochondrial DNA control region sequences. As a result, a total of 30 variable sites were detected in the 458 bp segment of the control region among 330 individuals from 11 localities, and 41 haplotypes were defined. Samples in the China coast present a high level of genetic diversity, with the values of haplotype diversity ranged from 0.674 to 0.860, and nucleotide diversity from 0.820% to 1.502%. Pairwise *F_ST_* statistics showed a moderate genetic divergence (−0.027 to 0.384) among samples from different geographical locations. Median-joining network analysis revealed a similar pattern of phylogeographic structure in samples from Ningbo and Dongxing although they were far apart. Therefore, joint influences of dispersal capability, spatial distance, ocean current and geographic segregation on the formation of the present population structure in *P. chinensis* was proposed. The results of the present study would be helpful for the sustainable utilization and management of this species.

## Introduction

The genetic background of fish species could facilitate the identification of appropriate management units, and a better understanding of this is, therefore, essential for resource recovery and delineation and monitoring of fishery management (Ward, 2000; Waples et al. 2008; Shui et al. 2009). Genetic structure of marine organisms may be influenced by many factors, such as currents, reproductive strategy and larval transport potential (Avise, 2000; Liu et al. 2009). And factors promoting lineage turnover and shallow population structure may take marine fishes vulnerable to overfishing and environmental change. A lack of background information on population structure had been reported to lead to local over-fishing and ultimately to severe declines (Roldán et al. 2000).

The Chinese pomfret *Pampus chinensis* is a coastal teleost species belonging to the *Pampus* family (Perciformes, Stromateoidei, Stomateidae). It has a wide distribution in the East China Sea and the South China Sea with a possible wider distribution from the Indian Ocean to eastern Indonesia. In China, it is common on the coast of Fujian, Guangdong and Guangxi province and viewed as a commercially important fish species. In recent decades, however, decline due to marine pollution and overfishing was found in this marine species (Zheng et al. 2003; Siyal 2013). To date, genetic background of *P. chinensis* is very limited (Cui et al. 2010; Sun et al. 2012; Sun and Tang 2018; Li et al. 2019), and limited information was known about its genetic structure and factors that influence it. Mitochondrial DNA (mtDNA) is proved to be an ideal marker for population genetic study, among which the control region gene has been reported to be highly sensitive in detecting population genetic structure and genetic diversity, and hence has been applied to population studies in various fish species, such as *Schizothorax prenanti*, *Scomberomorus niphonius*, *Auxis thazard, Katsuwonus pelamis*, *P. argenteus*, *Thunnus albacares*, etc. (Song et al. 2008; Shui et al. 2009; Kumar et al. 2012; Menezes et al. 2012; Sun et al. 2013; Kunal et al. 2013). In the present study, control region sequence was employed to evaluate the genetic variation and population structure of *P. chinensis* along the China coast. Meanwhile, samples from Indonesia region were also analyzed to explore the genetic divergence of *P. chinensis* between those from the China coast and other region. This study would provide valuable information not only for understanding the population genetic structure and its influence factors in *P. chinensis* but also for fishery management, conservation and sustainable exploitation of this species.

## Materials and methods

**Figure 1.**
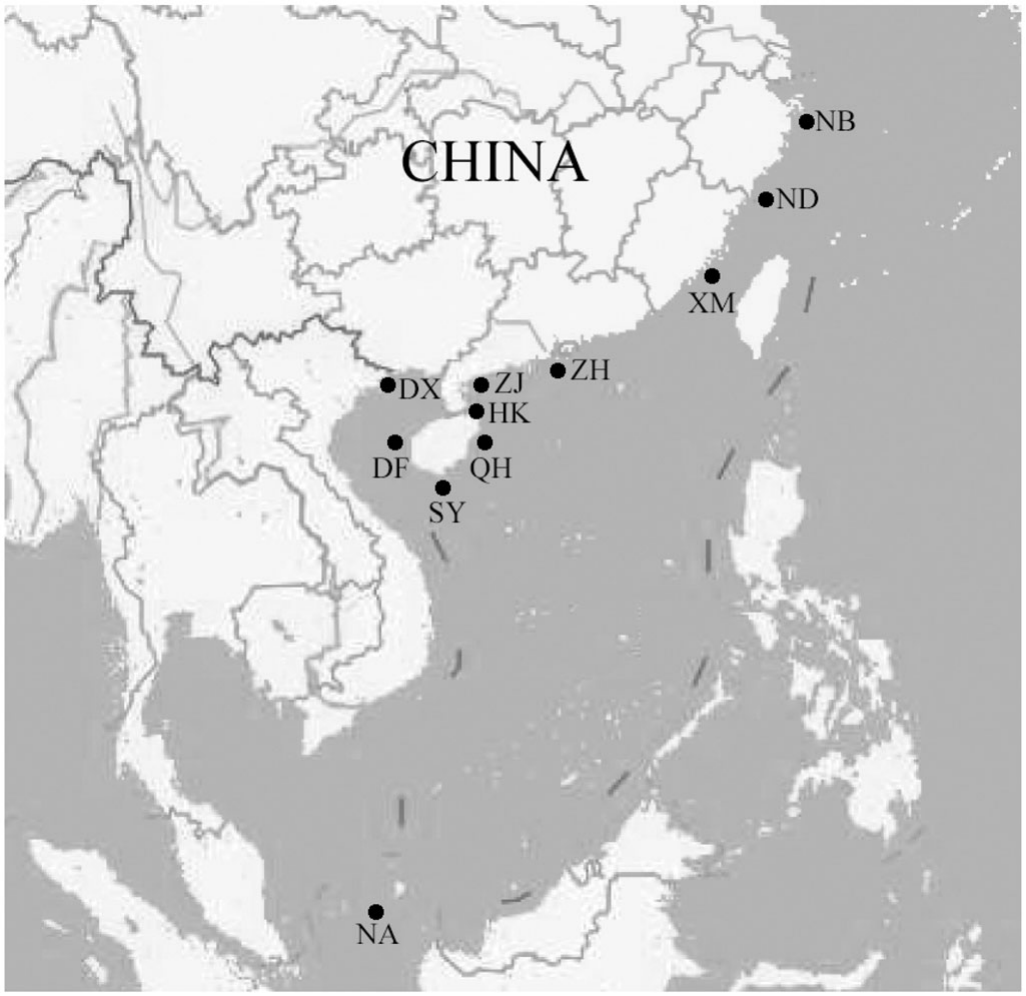
Locations of sample collection in present study. NB. Ningbo, the East China Sea; ND. Ningde, the East China Sea; XM. Xiamen, the East China Sea; ZH. Zhuhai, the South China Sea; ZJ. Zhanjiang, the South China Sea; HK. Haikou, the South China Sea; QH. Qionghai, the South China Sea; SY. Sanya, the South China Sea; DF. Dongfang, the South China Sea; DX. Dongxing, the South China Sea; NA. Natuna (Indonesia).

A total of 300 samples of *P. chinensis* were collected from 10 localities along the China coast (Figure 1 and Table 1). To facilitate the analysis, sample locations were further divided into four groups according to their geographical distribution: group 1 contains populations from the East China Sea (Ningbo, Ningde, and Xiamen); group 2 represents populations between Taiwan Strait and Hainan Island where has no noticeable barriers (Zhuhai, Zhanjiang, Haikou, Qionghai, Sanya, and Dongfang); group 3 is segregated in the Beibu Gulf (Dongxing); group 4 are individuals from the Natuna (Indonesia) used to compare with samples in the China coast. Muscle samples from each individual were preserved in 95% ethanol for genomic DNA extraction.

**Table 1.** Summary statistics of control region molecular indices in *Pampus chinensis*.

Samples	Locality	*n* ^a^	Haplotypes	*Hd* ^b^	*π*^c^ (%)	*k* ^d^
NB	Ningbo	30	10	0.807 ± 0.058	1.502 ± 0.809	6.878 ± 3.329
ND	Ningde	30	11	0.860 ± 0.041	0.875 ± 0.501	4.007 ± 2.060
XM	Xiamen	30	12	0.860 ± 0.048	1.043 ± 0.583	4.775 ± 2.400
ZH	Zhuhai	30	7	0.832 ± 0.037	0.820 ± 0.473	3.752 ± 1.946
ZJ	Zhanjiang	30	9	0.821 ± 0.050	0.937 ± 0.531	4.292 ± 2.186
HK	Haikou	30	6	0.745 ± 0.040	0.928 ± 0.527	4.248 ± 2.167
QH	Qionghai	30	4	0.674 ± 0.050	1.009 ± 0.567	4.621 ± 2.332
SY	Sanya	30	8	0.837 ± 0.037	0.903 ± 0.514	4.136 ± 2.117
DF	Dongfang	30	7	0.759 ± 0.059	0.887 ± 0.506	4.062 ± 2.084
DX	Dongxing	30	7	0.832 ± 0.034	1.260 ± 0.690	5.772 ± 2.841
NA	Natuna	30	3	0.248 ± 0.010	0.305 ± 0.214	1.398 ± 0.882

^a^Sample size.

^b^Haplotype diversity.

^c^Nucleotide diversity.

^d^Mean number of pairwise differences.

Genomic DNA was isolated from muscle tissue using TIANamp Genomic DNA kit (Tiangen, Beijing) following the introduction. Extracted DNA was checked using 1.5% agarose gel electrophoresis and then stored at −20 °C for PCR amplification. The control region partial sequence was amplified using the primer pair L15923 (5′-TTA AAG CAT CGA TCT TGT AA-3′) and H16500 (5′-GCC CTG AAA TAG GAA CCA GA-3′) (Inoue et al. 2000; Watanabe et al. 2002). Polymerase chain reaction (PCR) was performed in a 25 μl reactions mixture containing 1 × PCR buffer, 0.2 mM dNTPs, 1.5 mM MgCl_2_, 0.2 mM of each primer, 100 ng templates, and 1.0 unit Taq DNA polymerase (Tiangen, Beijing). The PCR was carried out under the following conditions: initial denaturation at 94 °C for 4 min, 35 cycles of 1 min at 94 °C for denaturation, 1 min at 55 °C for annealing, and 1 min at 72 °C for extension, with a final extension at 72 °C for 10 min. After separated by a 1.5% agarose gel and purified using the DNA Gel Extraction Kit (Shanghai Biotech Inc., Shanghai), the target PCR product was then sequenced in both directions using ABI 3730 automated DNA sequencer.

MtDNA sequences were edited and aligned using DNASTAR 7.1 software packages. Polymorphic site, number of haplotype, haplotype diversity (*Hd*), nucleotide diversity (*π*), and average number of pairwise nucleotide differences (*k*) were calculated in Arliquin v.3.01 (Excoffier et al. 2005). Median-joining network analysis of phylogenetic relationship among haplotypes was performed using Network 10.0 (Bandelt et al. 1999). The demographic history patterns of *P. chinensis* populations was estimate by neutrality Tajima’s *D* test, Fu’s *FS* test and goodness-of-fit test using Arliquin v.3.01. In addition, analysis of molecular variance components (AMOVA) (Excoffier et al. 1992) and the fixation index *F*_ST_ were carried out to evaluate the genetic differentiation within and between locations.

## Results

MtDNA control region sequences with a length of 458 base pairs (bp) were amplified and sequenced from 330 *P. chinensis* individuals, which defined 41 haplotypes (Genbank No: KY407441 - KY407481) with 30 polymorphic sites (Table 1). Of the 41 haplotypes, 28 were unique in corresponding locations and 13 were shared by different locations. Samples from Xiamen (XM) had the highest haplotype number (12) in all of the locations analyzed. The average values of pairwise differences among locations are ranged from 3.189 to 8.273, and the corrected average of pairwise difference ranges from −0.003 to 4.364 (Table 2). Values of haplotype diversity (*Hd*) for samples from the China coast ranged from 0.674 to 0.860, and values of nucleotide diversity (*π*) ranged from 0.820% to 1.502% (Table 1). But, samples from Natuna showed a low level of both *Hd* (0.248) and *π* (0.305%).

**Table 2.** Pairwise genetic differences for *P. chinensis*.

	NB	ND	XM	ZH	ZJ	HK	QH	SY	DX	DF	NA
NB	6.878	7.446**	7.863**	7.609**	7.880**	8.010**	8.142**	8.033**	6.367	7.740**	7.520**
ND	2.003**	4.007	4.556	4.564*	4.642*	4.880**	4.884*	5.033**	7.657**	4.462*	3.189**
XM	2.037**	0.165	4.775	4.298	4.502	4.593	4.667	4.618	7.981**	4.360	4.247**
ZH	2.294**	0.685**	0.035	3.752	3.958	3.986	4.200	3.889	7.633**	3.867*	4.702**
ZJ	2.295**	0.493*	−0.031	−0.064	4.292	4.193	4.462	4.200	7.924**	4.067	4.576**
HK	2.447**	0.752**	0.082	−0.014	−0.077	4.248	4.467	4.124	8.073**	4.081	4.878**
QH	2.393**	0.571*	−0.031	0.014	0.006	0.034	4.621	4.458	8.273**	4.329	4.649**
SY	2.526**	0.962**	0.163	−0.055	−0.014	−0.068	0.080	4.134	8.038**	4.096	5.273**
DX	0.041	2.767**	2.708**	2.871**	2.892**	3.063**	3.077**	3.084**	5.722	7.807**	7.849**
DF	2.270**	0.428*	−0.058	−0.038	−0.110	−0.074	−0.012	−0.003	2.889**	4.062	4.269**
NA	3.382**	0.487**	1.160**	2.128**	1.731**	2.055**	1.640**	2.507**	4.364**	1.539**	1.398

Above diagonal: average number of pairwise differences between locations (PiXY). Diagonal elements: average number of pairwise differences within locations (PiX). Below diagonal: corrected average number of pairwise difference (PiXY − (PiX + PiY)/2).

*p < 0.05.

**p < 0.01.

**Table 3.** Fixation index considering genetic distances (*F_ST_*) between samples of *P. chinensis* in different locations.

	NB	ND	XM	ZH	ZJ	HK	QH	SY	DX	DF	NA
NB											
ND	0.269**										
XM	0.259**	0.036									
ZH	0.301**	0.150**	0.008								
ZJ	0.291**	0.106*	−0.007	−0.016							
HK	0.305**	0.154**	0.018	−0.004	−0.018						
QH	0.294**	0.117**	0.007	0.003	0.001	0.008					
SY	0.314**	0.191**	0.035	−0.014	−0.003	−0.016	0.018				
DX	0.007	0.361**	0.339**	0.376**	0.365**	0.379**	0.372**	0.384**			
DF	0.293**	0.096*	−0.013	−0.010	−0.027	−0.018	−0.003	−0.001	0.370**		
NA	0.450**	0.153**	0.273**	0.452**	0.378**	0.421**	0.353**	0.475**	0.549**	0.361**	

*p < 0.05.

**p < 0.01.

Genetic structure of *P. chinensis* populations was analyzed by AMOVA (analysis of molecular variance components) and pairwise *F*_ST_ values. Results of AMOVA indicated that 76.43% of the total genetic variation occurred within the same locations, and 14.25% of the variation occurred among four different geographical groups (Table 4). Pairwise *F*_ST_ values ranged from 0.007 to 0.549. The statistics showed moderate genetic divergences among locations in different geographical groups. The average values of pairwise differences among locations are from 3.189 to 8.273 (Table 2). Significant genetic differentiation was found between samples of Ningbo, Dongxing or Natuna and other locations (*p <* 0.05). As for the other locations, no significant difference was found among samples from the same group. In addition, samples from Xiamen showed no significant genetic difference with those in group 2, which suggested a shallow genetic divergence among them.

**Table 4.** Analysis of molecular variance (AMOVA) of *P. chinensis* samples in different locations.

Source of variation	d.f.	Sum of squares	Variance component	Percentage of variation	Fixation indices
Among groups	3	102.524	0.266 Va	9.32	*F*_CT_^a^ = 0.093
Among locations within groups	7	100.540	0.406 Vb	14.25	*F*_SC_^b^ = 0.157**
Within locations	319	695.133	2.179 Vc	76.43	*F*_ST_^c^ = 0.236**
Total	329	898.197	2.851		

^a^Among group variations.

^b^Intra-group variations among locations.

^c^Total variance.

**p < 0.01.

**Figure 2.**
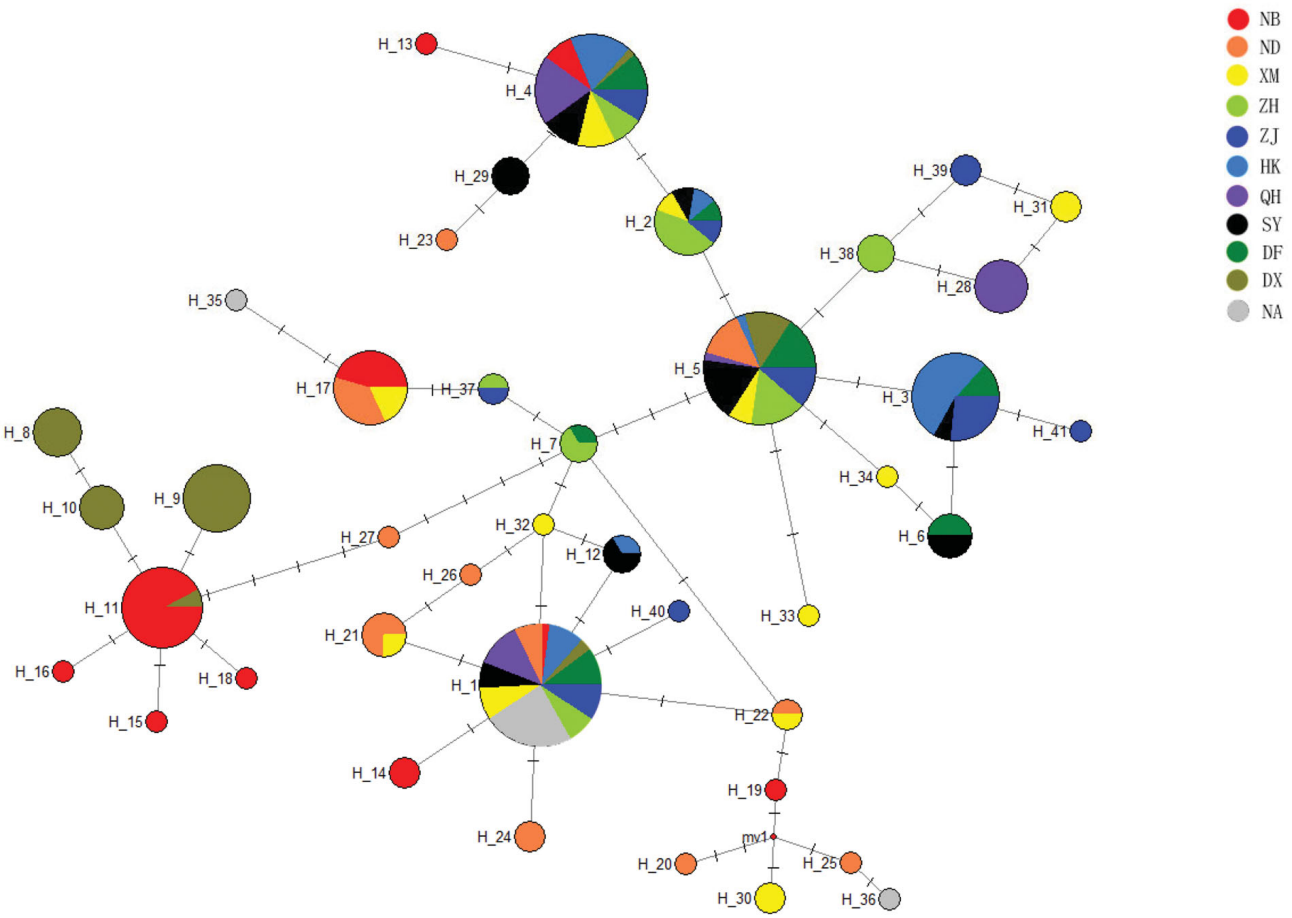
Median-joining network for 41 haplotypes of *P. chinensis*. Circle sizes are proportional to the frequencies of the respective haplotypes. The horizontal lines above the line between two haplotypes indicate mutated position. The median vectors indicate undiscovered haplotypes in the present study. Different colors indicate different geographic locations. NB. Ningbo, the East China Sea; ND. Ningde, the East China Sea; XM. Xiamen, the East China Sea; ZH. Zhuhai, the South China Sea; ZJ. Zhanjiang, the South China Sea; HK. Haikou, the South China Sea; QH. Qionghai, the South China Sea; SY. Sanya, the South China Sea; DF. Dongfang, the South China Sea; DX. Dongxing, the South China Sea; NA. Natuna (Indonesia).

To describe phylogenetic and geographical relationships among defined haplotypes, a network of all haplotypes with the median-joining method was constructed (Figure 2). The analysis revealed that all of the 41 haplotypes could be roughly divided into three branches which correspond to group 1, group 2 and group 3, respectively. Among all of the 41 haplotypes, 13 haplotypes were shared by different locations. An undiscovered haplotype was revealed with the median vectors in the present study. Besides, a distinct pattern of phylogeographic structure was found in samples of Ningbo and Dongxing. The neutral Tajima’s *D* and Fu’s *Fs* tests were shown in Table 5. Both the Tajima’s *D* test and the Fu’s *Fs* tests showed no significant values in all localities involved. Mismatch distribution analysis was also constructed for each location (Table 5). The raggedness index (Rg) ranged from 0.038 to 0.608 for 11 locations. The sum of the square deviations (SSD) ranged from 0.020 to 0.248. Therefore, a sudden population expansion model could not be deduced.

**Table 5.** Neutral test and goodness-of-fit test for *P. chinensis* samples in different locations.

Regions	Tajima’ *D*		Fu’s *Fs*		Goodness-of-fit test						
	D	*p*	*Fs*	*p*	*τ* ^a^	*θ*_0_^b^	*θ*_1_^c^	SSD^d^	*p*	Rg^e^	*p*
NB	1.263	0.907	1.710	0.789	11.443	0.000	19.172	0.077	0.010	0.131	0.020
ND	−0.227	0.441	−1.104	0.328	6.404	0.000	8.443	0.034	0.120	0.079	0.090
XM	0.386	0.680	−1.139	0.326	7.658	0.002	10.793	0.020	0.180	0.045	0.270
ZH	1.540	0.954	1.684	0.795	7.750	0.000	6.527	0.026	0.310	0.038	0.610
ZJ	1.017	0.870	0.586	0.664	8.137	0.002	8.188	0.035	0.210	0.056	0.370
HK	3.231	0.918	2.704	0.996	8.629	0.002	8.855	0.095	0.040	0.170	0.000
QH	2.630	0.996	6.694	0.983	9.000	0.000	13.320	0.248	0.020	0.608	0.000
SY	1.575	0.938	1.215	0.720	8.518	0.000	6.895	0.032	0.330	0.083	0.230
DF	1.930	0.974	2.003	0.812	8.305	0.002	7.617	0.062	0.070	0.153	0.040
DX	1.458	0.936	3.670	0.925	11.107	0.000	9.324	0.053	0.110	0.126	0.040
NA	−1.413	0.068	2.598	0.893	3.000	0.000	0.181	0.055	0.040	0.594	0.540

^a^Units of mutation time.

^b^*θ* before population growth.

^c^*θ* after population growth.

^d^The sum of the square deviations.

^e^Raggedness index.

## Discussion

Insight into genetic diversity and population genetic structure are essential for resource recovery and fishery management in fishes (Waples et al. 2008). In recent years, the study on population genetic of fish has attracted considerable interest (Sun et al. 2013; Kunal et al. 2013; Sun and Tang 2018; Li et al. 2019). The mtDNA control region has been proved to be useful for population studies of marine fishes (Liu et al. 2009; Shui et al. 2009; Sun et al. 2013).

*Pampus* are commercially important marine fishes that are widely distributed in the China coast and the Indo-West Pacific region (Almatar and James 2007; Sun et al. 2013). Sun et al. (2013) evaluated the genetic diversity of *P. argenteus* in the Indo-West Pacific region using control region sequences and reported high levels of genetic diversity in samples from Kuwait, Pakistan, India, and the South China Sea. Also, significant differences in different geographical groups were concluded. However, the population genetic background was still not clear in other *Pampus* species. Our previous study (Sun and Tang 2018) evaluated the *P. chinensis* along the China coast using mitochondrial DNA cytochrome b (cyt b) gene sequences. Results showed high level of haplotype diversity (0.540–0.828) and low level of nucleotide diversity (0.081–0.210%). And the low but significant F*st* statistics suggested genetic divergence among locations from different geographical regions. In addition, Li et al. (2019) compared *P. chinensis* samples between China and Pakistan using control region sequences, and distinct genetic heterogeneity was suggested. High level of both haplotype diversity (0.826–0.880) and nucleotide diversity (1.030–1.280%) was found in samples from the China coast. We deduced that the difference of genetic diversity for two studies was due to the different rate of genetic variation between cyt b and control region sequences.

Considering the advantage of control region sequences in assessing genetic structure of recently diverged or closely related populations, control region sequences were also employed in this study to assess the population structure of *P. chinensis* along the China coast. Results showed high level of both haplotype diversity (0.674–0.860) and nucleotide diversity (0.820–1.502%) in China samples, which was consistent with Li et al. (2019). High genetic variation patterns had also been reported in other fish species in the China coast, such as *Schizothorax prenanti*, *Pennahia argentata*, *Nibea albiflora* and *Cleisthenes herzensteini* (Song et al. 2008; Han et al. 2008a, 2008b; Xiao et al. 2011). High haplotype diversity level often suggests large, stable, effective population sizes. *P. chinensis* is known as a widely distributed and frequently observed species in China's coastal waters, and large population size may account for the high levels of haplotype diversity observed in this region.

The neutral Tajima’ *D* and Fu’s *Fs* test were employed to examine the historical demographic expansions of *P. chinensis*. Generally, significantly negative *D* and *Fs* values indicate either population expansion or purifying selection (Liu et al. 2009; Ren et al. 2017). In this study, both Tajima’s *D* and Fu’s *Fs* tests showed no significant difference in each location (*p* > 0.05). Meanwhile, statistical significance of SSD indicated that samples from ND, XM, ZH, ZJ, SY and DX were at equilibrium. Hence, a historical population expansion in those populations could not be deduced based on current data. Similar results were also found in *P. argenteus*, a sister species of *P. chinensis*. Sun et al. (2013) deduced that samples of *P. argenteus* from the South China Sea are at equilibrium and not in an expansion phase.

Marine fishes often show low levels of genetic differentiation among geographic regions. The general absent of noticeable barriers to dispersal in the marine environment effectively reduces heterogeneity among populations, often making it difficult to differentiate discrete populations. The population genetic structure of *P. chinensis* in this study seems conform to this pattern, especially samples with similar geographical distribution. Small and insignificant pairwise difference among locations was found in group 1 and group 2. Both the East China Sea and the South China Sea have wide and open coastline, a general absence of noticeable barriers in these marine environment may contribute the long-range gene exchange and the reduction of gene divergence among populations. This is consistent with conclusion obtained from other fish species in the China coast, such as *Scomberomorus niphonius*, *C. ectenes* and *P. argenteus* (Shui et al. 2009; Ma et al. 2010; Sun et al. 2013).

Genetic divergence of marine fish among different geographic localities could also be influenced by other factors, such as habitat difference, migration ability, and geographic segregation (Lourie et al. 2005; Liu et al. 2007). In China, the Taiwan Island exists between the East China Sea and the South China Sea, and it is connected to the mainland only by the narrow Taiwan Strait, which may influence gene exchange between these two sea regions. The moderate genetic divergence found between group 1 and group 2 confirmed that. Network results revealed that haplotypes could be roughly divided into three branches which correspond to group 1, group 2 and group 3, respectively. Likewise, the Beibu Gulf is isolated by Hainan Island, Leizhou Peninsula and the mainland, which may block gene exchange with the East China Sea and other part of the South China Sea. Geographical segregation of habitats may result in a higher genetic difference between it and the other two regions in the China Sea. Samples from Dongxing in the Beibu Gulf seems to fall into this case. In the meantime, the westward flow in the Qiongzhou Strait, causes a cyclonic gyre and the eastward flow causes an anti-cyclonic gyre instead. The outer flow has little effect on the current inside the gulf (Zu 2005). The existence of this currents may further block gene flow between marine fish inside and outside of the Beibu Gulf, which causes gene divergence between samples of Dongxing and other locations.

Besides, extrinsic forces such as ocean currents may also play an important role in transporting the larvae of marine organisms, which results in low gene divergence among populations. For example, the samples of Xiamen in group 1, showed no significant divergence with samples from both the East China Sea (Ningde) and the South China Sea (group 2).

## Conclusion

Insight into the genetic diversity and population structure would facilitate the fishery management and conservation of marine fish species. As an economically important fish species, the *P. chinensis* has a wide distribution in China. In the present study, genetic variation and population structure of *P. chinensis* along the China coast were evaluated and a combined influence of dispersal capability, spatial distribution, geographic segregation and ocean current on the formation of the present population structure in *P. chinensis* was assumed. The results would improve population genetic understanding and provide important implication for sustainable exploitation, fishery management and conservation of this species.

## Data Availability

The data that support the findings of this study are openly available in GenBank of NCBI at https://www.ncbi.nlm.nih.gov, reference number KY407441 - KY407481.
